# Mechanistic Insights Into Celastrol's Anti-Pyroptosis Effects in Osteoarthritis via SIRT2 Upregulation

**DOI:** 10.1155/mi/5676471

**Published:** 2025-09-08

**Authors:** Xiaotian Chen, Yining Song, Fan Zhang, Fangyan Hu, Zhenfei Ding, Jianzhong Guan

**Affiliations:** ^1^Department of Orthopaedics, The First Affiliated Hospital of Bengbu Medical University, Bengbu 233004, China; ^2^Anhui Province Key Laboratory of Tissue Transplantation, Bengbu Medical University, Bengbu 233004, China; ^3^School of Pharmacy, Bengbu Medical University, Bengbu 233030, China

**Keywords:** celastrol, NLRP3, osteoarthritis, pyroptosis, sirtuin 2

## Abstract

**Background:** Chronic inflammation and cell apoptosis are hallmark characteristics of osteoarthritis (OA), necessitating the development of novel therapeutic strategies. Celastrol (CSL) has emerged as a promising agent for OA treatment due to its anti-inflammatory properties, but the specific mechanism of action remains unclear.

**Methods:** This study utilized network pharmacology and in vivo experiments to elucidate how CSL modulates the SIRT2-NLRP3 axis in OA. Chondrocytes were treated with CSL to evaluate changes in SIRT2 expression and NLRP3 acetylation levels. OA rats were administered CSL to assess its therapeutic effects.

**Results:** Using network pharmacology and bioinformatics, SIRT2 and NLRP3 were identified as the primary therapeutic targets of CSL for OA. In vitro experiments demonstrated that CSL significantly reduced the levels of inflammatory markers and pyroptosis-related proteins, such as GSDMD-N, in TC28a cells and primary rat chondrocytes (RCs) induced by lipopolysaccharide (LPS) and nigericin (Nig). CSL upregulated SIRT2 expression, decreased NLRP3 acetylation, and promoted anti-inflammatory cytokine expression (IL-4 and IL-10), thereby, reducing inflammation and pyroptosis in chondrocytes. Notably, SIRT2 knockdown reversed CSL's anti-inflammatory and anti-pyroptosis effects. In vivo, CSL significantly alleviated OA symptoms in rats by modulating the SIRT2/NLRP3 pathway.

**Conclusion:** CSL exerts its anti-inflammatory effects in OA by targeting the SIRT2-NLRP3 axis to inhibit chondrocyte pyroptosis. These findings underscore the potential of CSL as a therapeutic agent for mitigating OA progression and offer new insights into its molecular mode of action.

## 1. Introduction

Osteoarthritis (OA) is a common and highly disabling chronic joint condition marked by cartilage breakdown, periarticular bone proliferation, synovial inflammation, and joint dysfunction [1–3]. With the global aging population and increasing obesity issues, the incidence of OA is on the rise, becoming a significant health concern affecting the quality of life for millions of people worldwide [4–6]. The pathogenesis of OA is related to the disruption of the delicate balance between joint tissue repair and destruction caused by mechanical stress and inflammatory cytokines [7]. Current treatments for OA primarily include nonsteroidal anti-inflammatory drugs (nonsteroidal antiinflammatory drugs, NSAIDs), intra-articular corticosteroid injections, hyaluronic acid, and joint replacement surgery [8–10]. However, these treatments mostly provide temporary symptom relief and are challenging to reverse or halt the progression of the disease [11–13]. Furthermore, prolonged use of NSAIDs and similar medications may lead to severe side effects such as gastrointestinal bleeding and renal impairment [14, 15]. Thus, new and more effective therapeutic approaches are urgently needed to improve OA treatment and enhance the quality of life for affected individuals [16–18].

Celastrol (CSL) is a natural pentacyclic triterpenoid compound derived from the traditional Chinese herb *Tripterygium wilfordii*, renowned for its anti-inflammatory, antioxidant, and immunomodulatory properties [19–21]. CSL has gained attention for its broad bioactivity and therapeutic potential across various disease models [20, 22]. For instance, in conditions like rheumatoid arthritis, autoimmune hepatitis, and neurodegenerative diseases, CSL has demonstrated promising therapeutic potential. Some studies indicate that CSL may be beneficial for treating OA. For instance, CSL enhances antioxidant capacity through activation of nuclear factor erythroid 2-related factor 2 (NRF2), preventing the progression of OA in mice [23]. Another study suggests that CSL may improve OA by modulating the TLR2/NF-κB signaling axis [24]. However, the precise mechanism of CSL in OA remains largely unknown, necessitating further investigation to identify its targets and clarify its potential mechanisms in OA treatment [25].

Despite the availability of multiple treatment options for OA, including NSAIDs, intra-articular corticosteroids, hyaluronic acid injections, and joint replacement surgery [26–29], the clinical management of OA remains challenging. Current treatments primarily focus on symptom relief rather than effectively halting disease progression. Moreover, prolonged use of NSAIDs can lead to gastrointestinal and cardiovascular complications, while corticosteroid injections, although effective in providing temporary symptom relief, have limited long-term benefits [30]. In recent years, biologic agents targeting IL-1 and TNF-α have been considered promising new therapies, but their high cost limits widespread clinical application. Consequently, there is an increasing demand for alternative treatments that provide both anti-inflammatory and chondroprotective benefits. Recent research suggests that targeting inflammasomes, oxidative stress, and autophagy pathways may offer new therapeutic strategies for OA. CSL, a natural pentacyclic triterpenoid, has garnered attention for its potent anti-inflammatory and immunomodulatory properties. However, the precise molecular mechanisms through which CSL exerts its therapeutic impact on OA remain poorly understood. This study aims to bridge this knowledge gap by elucidating the regulatory role of CSL in OA, particularly through its modulation of the SIRT2/NLRP3 signaling pathway.

This study utilized network pharmacology and bioinformatics methods to comprehensively investigate the potential targets of CSL and its mechanisms of action in OA treatment. Through this research, we aim to provide new insights into the potential mechanisms of CSL in OA treatment and offer novel molecular targets and therapeutic strategies for OA. The clinical significance lies in offering OA patients a novel treatment approach based on natural compounds, which may reduce the side effects of current treatment methods, improve treatment efficacy, and ultimately enhance patients' quality of life.

## 2. Materials and Methods

### 2.1. Retrieval of Information on Targets Related to CSL and OA

Using “celastrol” as a keyword, we conducted a literature search to obtain a list of targets associated with CSL. Simultaneously, the CTD database (http://ctdbase.org/) was accessed to gather target information for CSL. The retrieved target information was integrated, and duplicates were removed to generate a unique list of CSL targets. Information on targets related to OA was further obtained by accessing the GeneCards (https://www.genecards.org/) and CTD (http://ctdbase.org/) databases using the keywords “osteoarthrosis” and “degenerative_osteoarthrosis.” A Venn diagram was created using the venn.diagram function in the R software package VennDiagram to identify shared targets between CSL and OA, ensuring target specificity.

### 2.2. Retrieval of Bulk RNA Transcriptomic Data

The Gene Expression Omnibus (GEO) database (https://www.ncbi.nlm.nih.gov/geo/) was accessed to download transcriptomic datasets related to OA. The keyword “osteoarthritis” was typed in the search box and the most relevant dataset, GSE117999, was retrieved. This dataset consisted of 24 samples from *Homo sapiens*, including 12 samples of cartilage tissue from OA patients and 12 control samples. The R software was used for data formatting conversion and initial quality control processes on the downloaded data. To ensure the reliability of the data, stringent quality control standards were applied, including the removal of low-quality samples and batch effect correction.

### 2.3. Analysis of Differentially Expressed Genes (DEGs)

Differential expression analysis between OA and control samples was conducted using R (version 4.2.2) and the Bioconductor package edgeR (version 3.38.1). Following quality control and normalization, a GLM fitting model was applied to account for confounders, improving the analysis accuracy. The significance threshold was set as an adjusted *p*-value less than 0.05 to identify differential expression, reducing false positives. To better visualize these DEGs, a volcano plot was created using R software to display the upregulated and downregulated genes intuitively. To verify the reliability of the DEGs, sensitivity analysis was performed, including adjusting *p*-value thresholds and conducting repeated analysis to ensure the stability of the results.

### 2.4. Construction of Protein–Protein Interaction (PPI) Network

Based on the shared targets between CSL and OA, the PPI network of target genes was constructed utilizing the STRING online analysis platform (version 11.0). The species “*Homo sapiens*” was selected in the platform with a threshold set at medium confidence (0.400). Subsequently, interaction information was downloaded, and the PPI network was optimized using the Cytoscape software (version Cytoscape_v3.10.2) for clearer visualization. Differential expression genes were incorporated as node attributes. The topological coefficients of all nodes with multiple neighbors in the PPI network were calculated with the assistance of NetworkAnalyzer. To enhance the credibility of the PPI network, the network's topology was further analyzed to identify highly connected core targets, and the biological significance of these targets in OA was validated.

### 2.5. GO/KEGG Enrichment Analysis

Gene Ontology (GO) and Kyoto Encyclopedia of Genes and Genomes (KEGG) enrichment analysis for CSL target genes and DEGs was conducted using the “clusterProfiler” package (version 4.6.2), along with “org.Hs.eg.db” and “enrichplot.” Enrichment focused on biological processes (BP), cellular components (CC), molecular functions (MF), and relevant signaling pathways. A significance threshold of *p*  < 0.05 was applied, and false discovery rate (FDR) correction was used to control for false positives. Analyses were repeated to ensure robustness.

### 2.6. Molecular Docking to Validate the Interaction Between CSL and Target Proteins

The three-dimensional structure of CSL was obtained from the PubChem database (https://pubchem.ncbi.nlm.nih.gov/). “Celastrol” was entered into the search box to retrieve its chemical structure information, and the SDF format file of the three-dimensional structure was downloaded for subsequent analysis. The protein structures of key target proteins involved in OA interaction with CSL were retrieved from the Protein Data Bank (http://www.rcsb.org/).

Prior to docking, the protein database (PDB) file was preprocessed using PyMOL (version 2.4) to remove water molecules from the protein structure and add hydrogen atoms in AutoDock. For molecular docking analysis, docking parameters for both the target protein and CSL were prepared using AutoDock Vina software (version 4.2.6). A semi-flexible docking approach was employed, with a semi-empirical energy function used to interpret the docking results. The lowest binding free energy was selected as the optimal docking conformation, and the results were visualized using PyMOL.

### 2.7. Isolation of Primary Chondrocytes From Adult Rats

Primary rat chondrocytes (RCs) were isolated from the femoral head cartilage of 6-week-old SD rats. Cartilage fragments were cut into 2–5 mm^3^ pieces and digested enzymatically with 0.2% type II collagenase (ST2303, Betotime) at 37°C for 6 h.

To validate the successful isolation of primary cells, chondrocytes were seeded into 35 mm diameter confocal culture dishes (FCFC016, Beyotime) and cultured until the cells reached 80% confluence. After removing the culture medium, 3% BSA (ST025, Beyotime) was added evenly to cover the cells, and they were sealed at ambient temperature for 30 min. Type II collagen antibody (PA5-99159, Thermo Fisher) was added to each well, and the cells were incubated overnight in a humidified environment at 4°C. The culture dishes were then washed three times on a decolorizing shaker. After drying, rabbit IgG (H + L) F(ab')2 fragments (4412, CST) were added to cover the cells. The nuclei were stained with DAPI (C1002, Beyotime), and the slides were sealed with an anti-fluorescence quenching polyvinyl alcohol sealing agent (P0126, Beyotime). The cells were then observed and imaged with a confocal microscope.

### 2.8. Cell Culture, Lentivirus Infection, and Grouping

RC and TC28a cells (SCC042, Sigma) were cultured in high-glucose DMEM medium containing 10% fetal bovine serum (FBS, 12483020, Gibco), 100 units/mL penicillin, and 100 μg/mL streptomycin (C0222, Beyotime) [31, 32], and maintained in a humidified incubator at 37°C with 5% CO_2_.

The cells were divided into five groups: control (CTR), lipopolysaccharide (LPS) + Nig, LPS + Nig + CSL, LPS + Nig + CSL + shNC, and LPS + Nig + CSL + shSIRT2. The CTR group received PBS treatment and no additional interventions. The NLRP3 inflammasome was activated by co-treatment with LPS and Nigericin (Nig). Specifically, cells were treated with 100 ng/mL LPS for 3 h, followed by 10 μM Nig (purchased from BOCSCI: 28380-24-7) induction for 1 h. In the LPS + Nig + CSL group, after treatment with LPS and Nig, cells were cultured in fresh medium containing 1 μmol/L CSL (BOCSCI, B0084-187070) for 24 h..

SIRT2 knockdown was performed via lentiviral transduction during the logarithmic growth phase of RC and TC28a cells using lentivirus from Shanghai GeneChem Co., Ltd. Cells (5 × 10^4^ cells/mL) were seeded in 6-well plates (2 mL/well) and incubated overnight at 37°C. Recombinant lentivirus (1 × 10^8^ TU/mL) was added, and cells were cultured for 48 h before subsequent treatments [33]. The LPS + Nig + CSL + shNC and LPS + Nig + CSL + shSIRT2 groups were transduced with control or SIRT2 knockdown lentivirus, respectively, followed by LPS, Nig, and CSL treatment as previously described.

### 2.9. Western Blot Analysis

Total protein was extracted using RIPA lysis buffer with PMSF (P0013B, Beyotime, Shanghai) and quantified by BCA assay (23225, Thermo Fisher Scientific, Rockford, IL, USA). Protein samples (50 μg) were mixed with 2 × SDS buffer, boiled at 100°C for 5 min, and subjected to SDS–PAGE gel electrophoresis. Proteins were transferred to a PVDF membrane via the wet transfer method. The membrane was blocked with 5% non-fat milk at ambient temperature for 1 h, then incubated overnight at 4°C with primary antibodies: NLRP3 (A5652, abclonal), IL-1β (A16288, abclonal), cleaved IL-1β (AF4006, affbiotech), caspase-1 (A16792, abclonal), cleaved caspase-1 (AF4005, affbiotech), SIRT2 (A0273, abclonal), GSDMD/GSDMD-N (A10164, abclonal), acetylated lysine (05-515, Sigma), and actin (AC026, abclonal). After three washes with TBST, the membrane was incubated with HRP-conjugated goat anti-rabbit IgG secondary antibody (ab97051, 1:2000, Abcam) for 1 h. Following additional washes with TBST, the membrane was treated with Pierce ECL detection reagent (32209, Thermo) and visualized using the Bio-Rad ChemiDoc XRS + imaging system.

### 2.10. Enzyme-Linked Immunosorbent Assay (ELISA) Biochemical Detection

Culture supernatants were centrifuged at 1500 × *g* for 15 min. Following the ELISA antibody manual, the following antibodies were used: IL-1β (human: E-HSEL-H0001; rat: E-EL-R0012), TNF-α (human: E-EL-H0109; rat: E-EL-R2856), IL-4 (human: E-EL-H0101; rat: E-EL-R0014), IL-10 (human: E-EL-H6154; rat: E-EL-R0016), SAA (human: E-EL-H2183; rat: E-EL-R3026), and CRP (human: E-UNEL-H0029; rat: E-UNEL-R0006). Each well received 100 μL of dilution buffer, sample, and standard, followed by incubation at 37°C for 90 min. Biotinylated detection antibody (100 μL) was added and incubated for 1 h at 37°C. After three washes (2 min each), 100 μL of HRP conjugate was added and incubated for 30 min. After washing five times, substrate reagent was added, and the reaction was terminated after 15 min at 37°C in the dark. Absorbance values at 450 nm were measured using the Epoch microplate spectrophotometer (Bio-Tek, Winooski, VT, USA), with each sample run in triplicate.

### 2.11. Flow Cytometry

To assess cell apoptosis, treated cells were collected and centrifuged at 1000 × *g* for 5 min in pre-chilled PBS, with the wash step repeated twice. Cells were resuspended in 500 μL of cold PBS and stained with FITC-conjugated anti-caspase-1 antibody (A16792, Abclonal). Additionally, cells with membrane permeability were labeled with propidium iodide (PI) (F10797, Thermo Fisher Scientific). The cells that underwent apoptosis were identified based on the detection of active caspase-1 and PI positivity (Caspase-1 + PI + population).

### 2.12. CCK-8 Assay

Cell viability was determined using the CCK-8 kit (C0037, Beyotime). RC and TC28a cells were seeded at 4 × 10^3^ cells/well in 96-well plates with 100 μL of culture medium. After 24 h, cells were treated according to the experimental design and incubated for an additional 20 h. Subsequently, 10 μL of CCK-8 solution was added to each well, and absorbance at 450 nm was measured after 120 min of incubation at 37°C [34].

To evaluate intracellular SIRT2 enzymatic activity, cell lysates were collected and assessed using ELISA kits specific for human (105739) and rat (ml058883) SIRT2 (Shanghai Enzyme-linked Biotechnology Co., Ltd.). SIRT2-specific antibodies were used to capture the enzyme from the lysates. A reaction mixture containing 1 mM NAD^+^ and an acetylated substrate was then added, and the samples were incubated at 37°C for 30–60 min. The absorbance of deacetylated products was measured at 450 nm using a microplate reader. Enzymatic activity was quantified based on a recombinant SIRT2 standard curve and normalized to total protein content [35].

Lactate dehydrogenase (LDH) release was measured to assess cytotoxicity. The culture supernatants were harvested and centrifuged at 300 × *g* for 5 min to remove debris. LDH activity was assessed using a commercial LDH assay kit (C0016, Beyotime). Briefly, supernatants were mixed with substrate solution containing lactate, NAD^+^, and tetrazolium salt, and incubated at 37°C for 30 min in the dark. The reaction was terminated by adding a stop solution (e.g., 1 N HCl), and absorbance was measured at 490 nm. LDH release (%) was calculated using the formula: LDH release (%) = (OD_exp_ − OD_background_/(OD_max_ − OD_background_) × 100, where OD_max_ is the value from fully lysed cells (positive control) [36].

### 2.13. Immunoprecipitation

In the exogenous Co-IP experiment, Flag-NLRP3 and HA-SIRT2 were co-transfected into HEK-293T cells. Cell lysis was performed using IP cell lysis buffer (P0013, BEYOTIME) enriched with 1% protease inhibitor (P1005, BEYOTIME) at 4°C for 30 min. Lysates were centrifuged at 12,000 × *g* for 10 min at 4°C, and the supernatants were incubated overnight at 4°C with 20 μL of magnetic beads (P2108, Beyotime) conjugated to anti-Flag antibody (AE061, Abclonal). Beads were washed five times with TBST and resuspended in 1 × loading buffer (P0015A, Beyotime), denatured at 95°C for 10 min, centrifuged at 12,000 × *g* for 1–2 min at 4°C, and subjected to immunoblotting.

For endogenous Co-IP, RC, or TC28a cells were lysed using IP lysis buffer. The lysate was incubated at 4°C for 12 h with 20 μL of magnetic beads and either NLRP3 primary antibody or IgG (AC042, ABCLONAL). The immunocomplexes were collected by centrifugation at 1500 rpm for 5 min at 4°C, washed five times with TBST, and analyzed by Western blotting.

### 2.14. Construction and Treatment Grouping of the OA Rat Model

This study used 6-week-old Sprague-Dawley (SD) rats (180–220 g, Slac: SD, Shanghai SLAC Laboratory Animal Co., Ltd.), housed in a specific pathogen-free (SPF) facility with controlled conditions (12 h light/dark cycle, 22 ± 2°C, 50% ± 10% humidity), with ad libitum access to food and water.

The OA rat model was established by intra-articular injection of monosodium iodoacetate (MIA). A total of 42 SD rats were randomly assigned to seven groups (*n* = 6 per group): sham group (sham surgery), model group (MIA-induced OA), model + CSL group (OA model treated with CSL), model + CSL + shNC group (OA model treated with CSL and control shRNA lentivirus), model + CSL + shSIRT2 group (OA model treated with CSL and SIRT2 knockdown), model + CSL + oeNC group (OA model treated with CSL and control overexpression lentivirus), and model + CSL + oeNLRP3 group (OA model treated with CSL and NLRP3 overexpression lentivirus). The OA model was induced by a single intra-articular injection of 3 mg/50 μL MIA (Sigma–Aldrich) into the left knee joint, while the sham group received an equal volume of PBS. Treatments were initiated 14 days after model induction [37]. From day 14 post-induction, 0.05 mL of CSL solution (20 μg/mL) was administered intra-articularly daily for 35 days, with PBS as a control [38]. Concurrently, on the first day of CSL treatment, every 4 days, 50 μL of 1 × 10^9^ pfu shSIRT2/shNC/oeNC/oeNLRP3 lentivirus was intra-articularly injected into the rat joint [39]. After 35 days of treatment, rats were euthanized by cervical dislocation following pentobarbital sodium anesthesia. Cartilage tissue and tibial subchondral bone were collected for analysis. Micro-computed tomography (micro-CT) was used to assess cartilage damage, H&E staining for cartilage injury, western blot and ELISA for inflammatory markers, and immunohistochemistry to detect NLRP3 and SIRT2 expression in cartilage.

### 2.15. Locomotor Distance Testing

Rats were placed in the corner of an open field test chamber measuring 40 cm × 40 cm × 30 cm (VersaMax Legacy Open Field, Omnitech Electronics) and allowed to freely explore the area for 5 min. The behavioral testing chamber was positioned inside a sound-attenuated box equipped with ambient lighting and a fan. Movement and location were monitored using the AccuScan Fusion activity system, which tracked beam breaks to calculate the total distance traveled and the time spent stationary at the center.

### 2.16. Standstill Time Assessment

Rats were placed in a 40 cm × 40 cm × 30 cm open field test chamber (VersaMax Legacy Open Field, Omnitech Electronics) devoid of sound and odor interference, and under dark conditions. Standstill time of the rats was measured through beam breaks within the AccuScan Fusion activity system for a continuous 5-min period.

### 2.17. Micro-CT Analysis

Articular cartilage tissue was fixed in 10% formalin (F5554, Sigma) overnight. Micro-CT (SkyScan 1172; Bruker) was employed to analyze the microstructure of the samples. For the animal study, 50 sagittal images of the tibial subchondral bone were collected and reconstructed into 3D models. A Gaussian filter (sigma = 0.8, support = 2) was applied for noise suppression. Bone mineral density (BMD) and bone volume/total volume (BV/TV) were analyzed as 3D structural parameters using built-in algorithms, with all analyses performed using the Scanco micro-CT software.

### 2.18. Hematoxylin and Eosin Staining

Following euthanasia, rat cartilage tissues were harvested and fixed in 4% formaldehyde solution. The samples were then embedded in paraffin, sectioned into 4 μm slices, and stained with H&E staining reagent (C0105M, Beyotime) for 1 min. After rinsing with tap water until clear, the tissues were counterstained with eosin solution for 15 s. Following counterstaining, the sections were transferred to 95% ethanol, further dehydrated in 100% ethanol, and cleared with xylene. The slides were mounted with neutral mounting medium (C1795, Sigma) and air-dried before microscopic imaging.

Histological scoring was independently performed by three blinded researchers using the OARSI grading system. Cartilage degeneration was assessed at four anatomical locations in the knee joint (medial and lateral tibial and femoral regions). The OA score was calculated as the product of the damage depth and surface injury scores, with the average OA score obtained from the four locations assessed by the researchers.

### 2.19. Immunohistochemistry

After euthanizing the rats, tissue samples were collected and fixed in 4% formaldehyde solution (P0147A, Beyotime). The tissues were embedded in paraffin, sectioned into 4 μm slices, and subjected to antigen retrieval by heating in a microwave with EDTA (P0085, Beyotime). The sections were then treated with 3% hydrogen peroxide to block endogenous peroxidase activity and incubated with goat serum (C0265, Beyotime) to block nonspecific binding. Primary antibodies, including NLRP3 (MA5-32255, Thermo Fisher Scientific) and SIRT2 (PA5-20487, Thermo Fisher Scientific), were applied and incubated overnight at 4°C. Afterward, biotinylated IgG secondary antibodies were applied, followed by incubation with anti-biotin peroxidase reagent for 20 min. Signal detection was performed using DAB reagent (P0202, Beyotime). The sections were scanned using the Pannoramic Midi scanner (3DHISTECH).

### 2.20. Statistical Analysis

Data were obtained from at least three independent experiments and presented as mean ± standard deviation (mean ± SD). Comparisons between two groups were conducted using unpaired two-tailed Student's *t*-tests. For comparisons among three or more groups, one-way ANOVA followed by Tukey's honestly significant difference (HSD) post hoc test was applied. For non-normally distributed data or unequal variances, the Mann–Whitney *U* test or Kruskal–Wallis *H* test was used. Statistical analysis was carried out using GraphPad Prism 9 (GraphPad Software, Inc.) and R programing language. A significance level of 0.05 was set for all tests, and a two-tailed *p*-value less than 0.05 was considered statistically significant.

## 3. Results

### 3.1. Screening of Potential Targets of Celastracol for Inhibiting the Inflammatory Progression of OA

**Figure 1:**
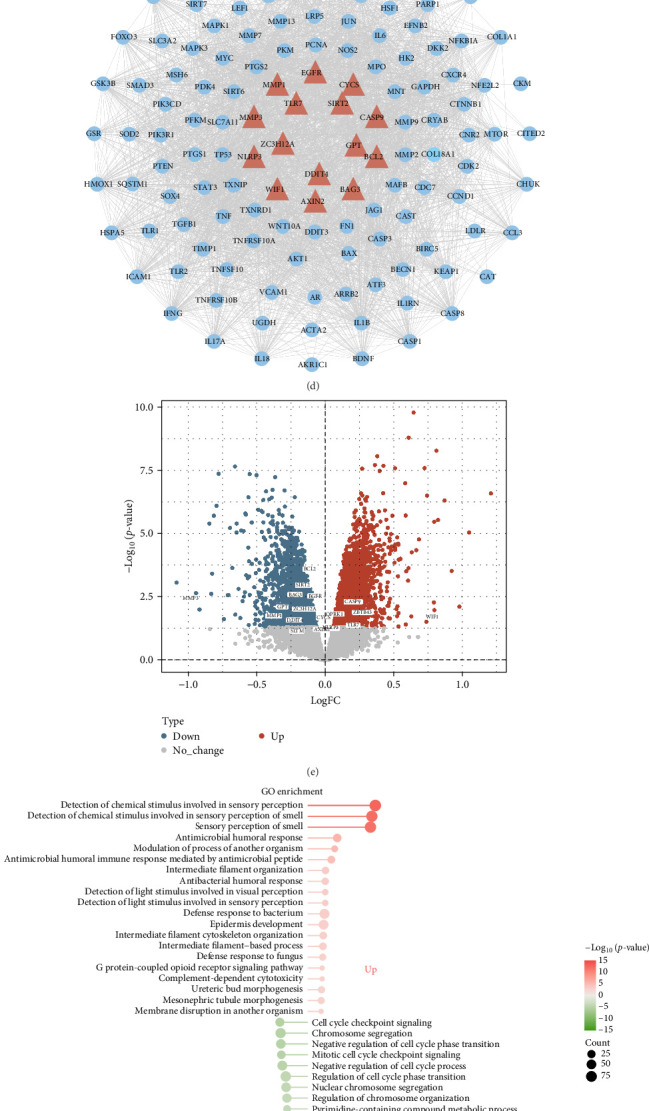
Preliminary screening of potential targets of CSL in inhibiting the inflammatory progression of OA. Note: (A) GO enrichment analysis of CSL targets. Blue, red, and yellow represent BP, CC, and MF categories, respectively. (B) KEGG pathway analysis of CSL targets, with darker colors indicating higher enrichment levels. (C) Integration of CSL and OA target data, identifying 135 relevant targets of CSL against OA. (D) PPI network of CSL-related targets against OA, with red triangles marking key targets; (E) volcano plot of DEGs, showing upregulation in red and downregulation in blue. (F) GO functional enrichment analysis of DEGs, with red indicating upregulation and green indicating downregulation. (G) KEGG enrichment analysis of DEGs, with red indicating upregulation and green indicating downregulation. (H) Venn diagram showing intersecting genes of DEGs, OA targets, and CSL targets.

The screening process of the network pharmacology analysis in this study is illustrated in Suppoting Information 1: Figure S1. First, we identified 230 unique target points related to potential interactions with CSL through literature searches and queries in the CTD database. Eliminating duplicates after integration, we proceeded with functional annotation and pathway analysis of these targets. The analysis revealed their involvement in responses to hypoxia, regulation of apoptotic signaling pathways, oxidative stress responses, and Wnt signaling pathways, as well as participation in cellular apoptosis, IL-17 signaling pathways, and HIF-1 signaling pathways (Figure 1A,B). Excessive oxidative stress leads to mitochondrial dysfunction, promoting chondrocyte destruction, including inflammation and cell apoptosis. The number of apoptotic chondrocytes in cartilage positively correlates with the degradation degree mediated by OA. This suggests that CSL plays a crucial role in influencing biological functions, especially cell apoptosis, which promotes the occurrence and development of OA [40, 41]. These findings provide a robust molecular basis for further exploring CSL's anti-OA effects.

Furthermore, we acquired 3661 OA-related target points from GeneCards and the CTD database. Through intersection analysis, we identified 135 potential therapeutic targets of CSL in anti-OA treatment (Figure 1C). Subsequently, we utilized the STRING database to build a PPI network of these therapeutic targets (Figure 1D). After removing disconnected nodes, the network comprised 131 nodes, with these genes playing vital roles in the pathogenic mechanism of OA. We also downloaded the transcriptome dataset GSE117999 related to OA from the GEO database and identified a total of 7900 DEGs in OA patients, including 4508 upregulated genes and 3392 downregulated genes. These genes exhibit significant differential expression between OA sample groups and healthy control groups (Figure 1E). Further, GO and KEGG enrichment analysis revealed that the upregulated DEGs are mainly involved in processes such as G protein-coupled receptor signaling pathways, complement-dependent cytotoxicity, tyrosine metabolism, and neuroactive ligand-receptor interactions. On the other hand, the downregulated genes are primarily enriched in negative regulation of the cell cycle, DNA replication, glycerophospholipid metabolism, autophagy, apoptosis, and the p53 signaling pathway (Figure 1F,G). These findings suggest differential apoptotic tendencies in osteoarthritic chondrocytes. Studies have indicated a positive correlation between cellular apoptosis in human OA tissue samples and the severity of cartilage destruction and matrix depletion [42–44]. The results of these enrichment analyses are highly consistent with the pathological mechanisms of OA, further validating the reliability of the screening results.

To further pinpoint the potential targets of CSL in treating OA, we conducted an intersection analysis among the DEGs, CSL targets, and OA-related targets, ultimately identifying 18 key targets (Figure 1H). Among these, 15 overlap with the PPI network nodes, visually highlighted in the PPI network of Figure 1D. These findings suggest that CSL may serve as a potential source for novel anti-OA medications. Through the involvement of these key targets, CSL may exert its anti-OA effects by modulating BP such as cell apoptosis and autophagy.

### 3.2. Celastral Modulates the SIRT2/NLRP3 Signaling Axis to Inhibit the Inflammatory Progression of OA

**Figure 2:**
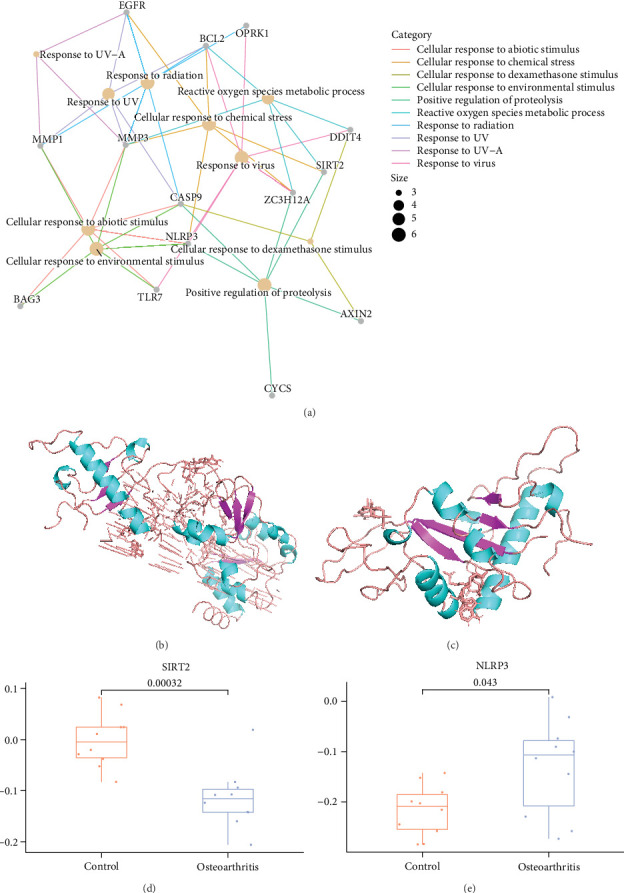
CSL regulates the SIRT2/NLRP3 signaling axis to counteract the inflammatory progression of OA. Note: (A) network diagram of the association between 18 key genes and their respective GO functional sets, with different colored nodes representing genes and functions, larger nodes indicating more genes enriched in them, and line colors indicating different functions. (B) Molecular interaction between the drug CSL and SIRT2. (C) Molecular interaction between the drug CSL and NLRP3. (D) Box plots showing the differential expression of SIRT2 in the GSE117999 dataset of OA (*N* = 12) and normal samples (*N* = 12), with red indicating control samples and blue indicating OA patient samples. (E) Box plots showing the differential expression of NLRP3 in the GSE117999 dataset of OA (*N* = 12) and normal samples (*N* = 12), with red indicating control samples and blue indicating OA patient samples.

To elucidate the molecular mechanism of CSL in combating OA, we conducted a thorough analysis of 18 key targets (Figure S1). We found that these targets are significantly enriched in processes related to protein hydrolysis, reactive oxygen species (ROS) metabolism, cellular response to chemical stress, and cellular response to non-biological stimuli, among other stress-related functions (Figure 2A). This indicates that these genes may be involved in cellular phenotype changes, including responses to oxidative stress and cell apoptosis [45]. For instance, the dysregulation of NLRP3 has been shown to promote extracellular matrix degradation and pyroptotic inflammation in osteoarthritic chondrocytes, thereby exacerbating OA symptoms [46]. Consequently, CSL could exert its anti-OA effects via pathways related to oxidative stress and the cellular response to non-biological factors.

The topological coefficient serves as an indicator of how closely a node is connected to others within the network. Using NetworkAnalyzer, we calculated the topological coefficients of 15 key targets in the PPI network and selected the top 10 key genes based on this analysis (Table S1) [47–49]. Subsequently, we examined the frequency of BP in which these genes are involved, identified the top five important targets, and then determined the intersection to obtain the five candidate genes for CSL's anti-OA effects: ZC3H12A, BCL2, NLRP3, SIRT2, and EGFR (Table S2). To validate the reliability of these candidate targets, we conducted molecular docking experiments. The results showed that CSL exhibited the lowest binding energy with SIRT2 and NLRP3, indicating that these two targets have high binding affinity.

When evaluating a high-quality drug target, it is essential to consider not only the target's significance in BP but also its accessibility to the drug. Bearing this in mind, we conducted molecular docking of the targets ZC3H12A, BCL2, NLRP3, SIRT2, and EGFR to further investigate the binding mechanisms of CSL with these core targets. A binding energy less than 0 kJ/mol indicates spontaneous binding and interaction between the molecule and the protein, with lower binding energies suggesting more stable molecular conformations [50]. The results showed that celastrol exhibited the lowest binding energies with SIRT2 (−13.26 kJ/mol) and NLRP3 (−10.63 kJ/mol), suggesting that these two proteins are the most likely targets of CSL. In contrast, the binding energies of ZC3H12A, BCL2, and EGFR were all higher than −10 kJ/mol and were therefore excluded (Table S3, Figure 2B,C). SIRT2 and NLRP3 exhibited significant expression differences between OA patients and normal samples. Specifically, SIRT2 was downregulated in OA samples, while NLRP3 levels were elevated (Figure 2D,E). Taken together, these findings suggest that SIRT2 and NLRP3 are potential target candidates for CSL in OA, indicating that CSL exerts its anti-OA effects by modulating the SIRT2/NLRP3 signaling pathway.

### 3.3. CSL Reduces Inflammation and Pyroptosis Levels in Chondrocytes

In our study, we selected the human chondrocyte cell line TC28a and primary RCs for experimental purposes. Since RC cells needed to be isolated from rats, we conducted immunofluorescence staining specifically targeting type II collagen, a chondrocyte-specific marker, to validate the isolation and assess its purity [51]. The results indicated that over 98% of the cultured cells exhibited positive staining for collagen II (Figure S2), indicative of their suitability for further experimentation.

**Figure 3:**
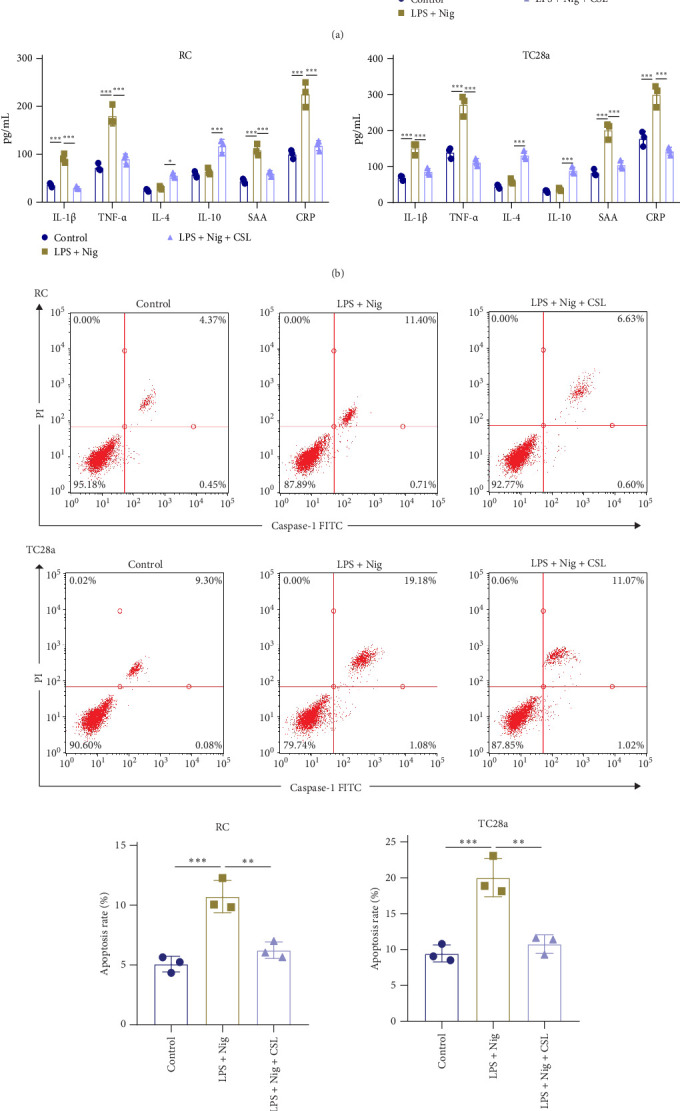
CSL reduces inflammation and pyroptosis levels in chondrocytes. Note: (A) Western blot analysis of the expression of key inflammatory proteins in RC and TC28a cells. (B) ELISA detection of the expression of inflammatory factors in RC and TC28a cells. (C) Flow cytometry analysis of apoptosis rate in RC and TC28a cells. (D) CCK8 assay to assess cell viability of RC and TC28a cells. Data are presented as mean ± SD (*n* = 3; ns *p*  > 0.05, *⁣*^*∗*^*p*  < 0.05, *⁣*^*∗∗*^*p*  < 0.01, *⁣*^*∗∗∗*^*p*  < 0.001, analyzed using ANOVA and Tukey's post hoc test).

Western blot analysis of RC and TC28a cells revealed that, when compared to the CTR group, the LPS + Nig treatment group exhibited markedly higher levels of inflammasome NLRP3 (RC: CTR vs. LPS + Nig, *p*  < 0.001; LPS + Nig vs. LPS + Nig + CSL, *p*  < 0.001; TC28a: CTR vs. LPS + Nig, *p*  < 0.001; LPS + Nig vs. LPS + Nig + CSL, *p*  < 0.001), downstream activated protein caspase-1 (RC: CTR vs. LPS + Nig, *p*  < 0.001; LPS + Nig vs. LPS + Nig + CSL, *p*  < 0.001; TC28a: CTR vs. LPS + Nig, *p*  < 0.001; LPS + Nig vs. LPS + Nig + CSL, *p*  < 0.001), pro-inflammatory cytokine IL-1β (RC: CTR vs. LPS + Nig, *p*  < 0.001; LPS + Nig vs. LPS + Nig + CSL, *p*  < 0.001; TC28a: CTR vs. LPS + Nig, *p*  < 0.001; LPS + Nig vs. LPS + Nig + CSL, *p*  < 0.001), and pyroptosis key protein GSDMD-N (RC: CTR vs. LPS + Nig, *p*  < 0.001; LPS + Nig vs. LPS + Nig + CSL, *p*  < 0.001; TC28a: CTR vs. LPS + Nig, *p*  < 0.001; LPS + Nig vs. LPS + Nig + CSL, *p*  < 0.001) [52]. Compared to the LPS + Nig group, the LPS + Nig + CSL group exhibited a notable reduction in the expression of these critical proteins (Figure 3A).

Flow cytometry analysis revealed that the pyroptosis rate was significantly higher in the LPS + Nig group compared to the CTR group, the LPS + Nig group showed increased expression of pro-inflammatory cytokines IL-1β and TNF-α (RC: IL-1β, *p*  < 0.001; TNF-α, *p*  < 0.001; TC28a: IL-1β, *p*  < 0.001; TNF-α, *p*  < 0.001), unchanged expression of anti-inflammatory cytokines IL-4 and IL-10 (RC: IL-4, *p*=0.8423; IL-10, *p*=0.7777; TC28a: IL-4, *p*=0.4962; IL-10, *p*=0.9201), and elevated expression of key inflammatory factors SAA and CRP (RC: SAA, *p*  < 0.001; CRP, *p*  < 0.001; TC28a: SAA, *p*  < 0.001; CRP, *p*  < 0.001). In contrast, the LPS + Nig + CSL group showed reduced expression of IL-1β and TNF-α (RC: IL-1β, *p*  < 0.001; TNF-α, *p*  < 0.001; TC28a: IL-1β, *p*  < 0.001; TNF-α, *p*  < 0.001), increased expression of IL-4 and IL-10 (RC: IL-4, *p*=0.0149; IL-10, *p*  < 0.001; TC28a: IL-4, *p*  < 0.001; IL-10, *p*  < 0.001), and reduced expression of SAA and CRP (RC: SAA, *p*  < 0.001; CRP, *p*  < 0.001; TC28a: SAA, *p*  < 0.001; CRP, *p*  < 0.001) (Figure 3B).

Flow cytometry analysis revealed that the pyroptosis rate was significantly higher in the LPS + Nig group relative to the CTR group (RC: *p*  < 0.001; TC28a: *p*=0.001), while the LPS + Nig + CSL group showed a marked reduction compared to the LPS + Nig group (RC: *p*=0.0029; TC28a: *p*=0.002) (Figure 3C).

CCK-8 assays demonstrated decreased cell viability in the LPS + Nig group compared to the CTR group (RC: *p*  < 0.001; TC28a: *p*  < 0.001), while the LPS + Nig + CSL group exhibited increased cell viability compared to the LPS + Nig group (RC: *p*=0.0027; TC28a: *p*  < 0.001) (Figure 3D).

In conclusion, CSL reduces both inflammatory and pyroptotic levels in humans and RCs.

### 3.4. CSL Inhibits Inflammatory Progression of OA via the SIRT2/NLRP3 Axis

Network pharmacology analysis reveals that SIRT2 is another highly reputable target of CSL. SIRT2, a pivotal deacetylation protein, and recent studies have found that SIRT2 can inhibit the activation of NLRP3 by deacetylating it, thereby, regulating inflammation and pyroptosis [53–55]. Transcriptomic analysis of OA highlights the differential expression of SIRT2 and NLRP3, suggesting that CSL may impede the inflammatory progression of OA through the SIRT2/NLRP3 axis.

**Figure 4:**
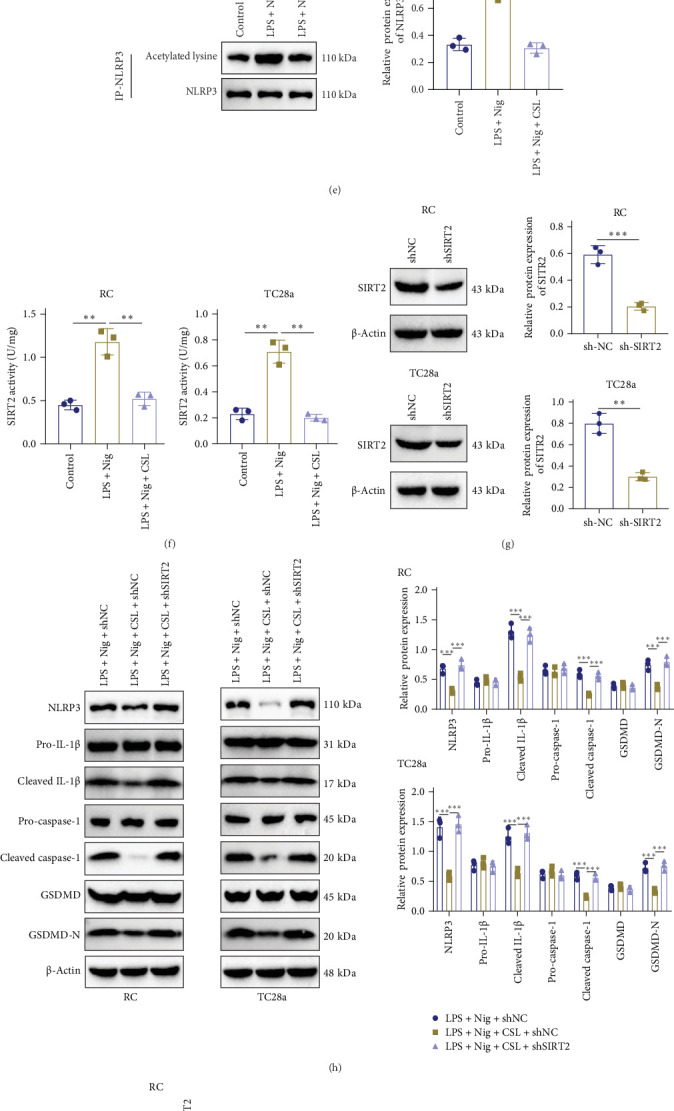
CSL inhibits the inflammatory progression of OA through the SIRT2/NLRP3 axis. Note: (A) Exogenous immunoprecipitation to detect the interaction between NLRP3 and SIRT2. (B) Endogenous immunoprecipitation to detect the interaction between NLRP3 and SIRT2 in RC and TC28a cells. (C) Western blot analysis of NLRP3 and SIRT2 protein levels in RC and TC28a cells. (D) Acetylation analysis of NLRP3 in RC and TC28a cells following SIRT2 knockdown. (E) Acetylation analysis of NLRP3 acetylation levels in RC and TC28a cells. (F) ELISA analysis of SIRT2 enzymatic activity in RC and TC28a cells. (G) Western blot analysis of knockdown levels of SIRT2 protein in RC and TC28a cells. (H) Western blot analysis of the expression of key inflammatory proteins in RC and TC28a cells with SIRT2 protein knockdown. (I) Acetylation analysis of NLRP3 acetylation levels in RC and TC28a cells with SIRT2 protein knockdown. (J) Flow cytometry analysis of pyroptosis rate in RC and TC28a cells with SIRT2 protein knockdown. (K) ELISA detection of the expression of inflammatory factors in RC and TC28a cells with SIRT2 protein knockdown. (L) CCK8 assay to assess cell viability of RC and TC28a cells with SIRT2 protein knockdown. (M) LDH release assay to measure LDH levels in RC and TC28a cells. Data are presented as mean ± SD (*n* = 3; *⁣*^*∗∗*^*p*  < 0.01, *⁣*^*∗∗∗*^*p*  < 0.001, analyzed using ANOVA and Tukey's post hoc test).

Exogenous and endogenous immunoprecipitation confirmed the interaction between NLRP3 and SIRT2 (Figure 4A,B). In RC and TC28a cells, western blot analysis showed that compared to the CTR group, the LPS + Nig group had increased expression of NLRP3 (RC: *p*  < 0.001; TC28a: *p*  < 0.001) and decreased expression of SIRT2 (RC: *p*  < 0.001; TC28a: *p*  < 0.001). In contrast, the LPS + Nig + CSL group showed decreased NLRP3 expression (RC: *p*  < 0.001; TC28a: *p*  < 0.001) and increased SIRT2 expression (RC: *p*  < 0.001; TC28a: *p*  < 0.001) relative to the LPS + Nig group (Figure 4C).

By assessing NLRP3 acetylation before and after SIRT2 silencing in both RC and TC28a cells, the results showed that NLRP3 acetylation was increased in the shSIRT2 group versus the shNC group (RC: *p*  < 0.001; TC28a: *p*  < 0.001) (Figure 4D). Acetylation also increased in the LPS + Nig group versus the CTR group (RC: *p*  < 0.001; TC28a: *p*  < 0.001) but was reduced in the LPS + Nig + CSL group compared to LPS + Nig (RC: *p*  < 0.001; TC28a: *p*  < 0.001) (Figure 4E). ELISA was performed to measure SIRT2 enzymatic activity in RC and TC28a cells. The results indicated that SIRT2 activity was decreased in the LPS + Nig group versus the CTR group (RC: *p*  < 0.001; TC28a: *p*  < 0.001) but increased in the LPS + Nig + CSL group compared to LPS + Nig (RC: *p*  < 0.001; TC28a: *p*  < 0.001) (Figure 4F).

To verify that CSL inhibits NLRP3 activation by upregulating SIRT2 and reducing NLRP3 acetylation, SIRT2 was knocked down in RC and TC28a cells (RC: *p*  < 0.001; TC28a: *p*  < 0.001) (Figure 4G). Western blot analysis showed that NLRP3, caspase-1, IL-1β, and GSDMD-N expression were significantly lower in the LPS + Nig + CSL + shNC group compared to LPS + Nig + shNC (RC: *p*  < 0.001; TC28a: *p*  < 0.001). However, these protein levels were increased in the LPS + Nig + CSL + shSIRT2 group compared to LPS + Nig + CSL + shNC (Figure 4H).

NLRP3 acetylation was reduced in the LPS + Nig + CSL + shNC group relative to the LPS + Nig + shNC group (RC: *p*=0.0015; TC28a: *p*=0.001), but increased in the LPS + Nig + CSL + shSIRT2 group compared to the LPS + Nig + CSL + shNC group (RC: *p*=0.0001; TC28a: *p*=0.0006) (Figure 4I).

Flow cytometry analysis revealed that the pyroptosis rate was lower in the LPS + Nig + CSL + shNC group relative to the LPS + Nig + shNC group (RC: *p*=0.0013; TC28a: *p*=0.0015), but higher in the LPS + Nig + CSL + shSIRT2 group compared to the LPS + Nig + CSL + shNC group (RC: *p*=0.0032; TC28a: *p*=0.0006) (Figure 4J).

ELISA results showed decreased IL-1β and TNF-α levels (RC: *p*  < 0.001; TC28a: *p*  < 0.001), increased IL-4 and IL-10 levels (RC: *p*  < 0.001; TC28a: *p*  < 0.001), and reduced SAA and CRP levels (RC: *p*  < 0.001; TC28a: *p*  < 0.001) in the LPS + Nig + CSL + shNC group compared to the LPS + Nig + shNC group. In contrast, the LPS + Nig + CSL + shSIRT2 group exhibited elevated IL-1β and TNF-α levels (RC: *p*  < 0.001; TC28a: *p*  < 0.001), reduced IL-4 and IL-10 levels (RC: IL-4 *p*=0.002; IL-10 *p*  < 0.001; TC28a: *p*  < 0.001), and increased SAA and CRP levels (RC: *p*  < 0.001; TC28a: CRP *p*=0.0027) (Figure 4K).

CCK-8 analysis of cell viability showed that the LPS + Nig + CSL + shNC group had increased cell viability compared to the LPS + Nig + shNC group (RC: *p*  < 0.001; TC28a: *p*=0.0003), while viability was decreased in the LPS + Nig + CSL + shSIRT2 group compared to the LPS + Nig + CSL + shNC group (RC: *p*  < 0.001; TC28a: *p*=0.0003) (Figure 4L). LDH release was lower in the LPS + Nig + CSL + shNC group compared to the LPS + Nig + shNC group (RC: *p*  < 0.001; TC28a: *p*  < 0.001), but higher in the LPS + Nig + CSL + shSIRT2 group compared to the LPS + Nig + CSL + shNC group (RC: *p*  < 0.001; TC28a: *p*  < 0.001) (Figure 4M).

These findings suggest that CSL inhibits the inflammatory progression of OA through the SIRT2/NLRP3 axis. Knocking down SIRT2 levels in both human and mouse chondrocytes can reverse the ability of *tripterygium wilfordii* to resist inflammation and pyroptosis.

### 3.5. Evaluation of the Therapeutic Effect of CSL on an OA Rat Model

**Figure 5:**
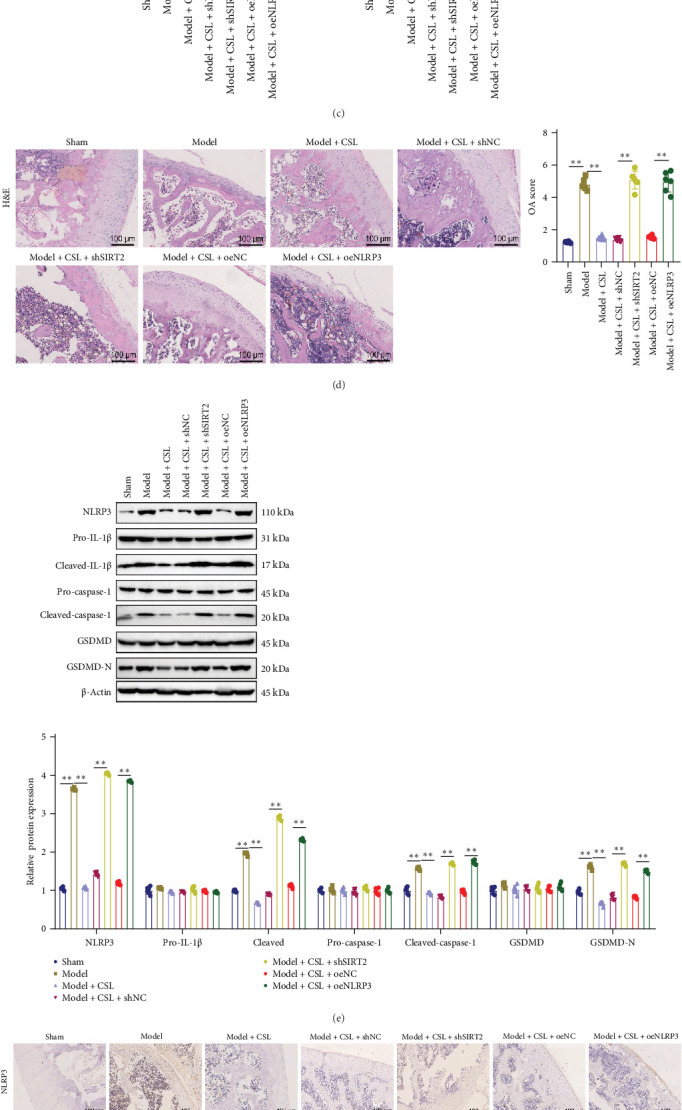
Evaluation of the therapeutic effect of CSL in an OA mouse model. Note: (A) Assessment of the 5-minute ambulatory distance in OA rats of each group. (B) Measurement of the standing time within 5 minutes in OA rats of each group. (C) Micro-CT scanning to detect 3D imaging of the medial tibial subchondral compartment in OA rats of each group, quantifying structural parameters of the tibial subchondral bone including BMD and BV/TV. (D) H&E staining was used to assess cartilage damage in the OA rat model. The cartilage thickness and the number of chondrocytes were reduced (indicated by arrows). (E) Western blot analysis of the expression of key inflammatory proteins in cartilage tissues of OA rats. (F) immunohistochemical analysis of the expression of NLRP3 and SIRT2 in cartilage tissues of OA rats. (G) ELISA detection of the expression of inflammatory factors in OA rats. Data are presented as mean ± SD (*n* = 6; *⁣*^*∗∗*^*p* < 0.01, analyzed using ANOVA and Tukey's post hoc test).

To investigate the role of tetrandrine in OA treatment and its mechanism via the SIRT2/NLRP3 axis, we induced OA in rats using MIA. Activity distance analysis revealed reduced activity in the model group compared to the sham group (*p*  < 0.01), with increased activity in the model + CSL group (*p*  < 0.01). However, locomotion was significantly reduced in the model + CSL + shSIRT2 and model + CSL + oeNLRP3 groups compared to their respective controls (*p*  < 0.01) (Figure 5A). Similarly, standing time was lower in the model group compared to the sham group (*p*  < 0.01), increased in the model + CSL group (*p*  < 0.01). Standing time decreased again in the model + CSL + shSIRT2 group compared to the model + CSL + shNC group (*p*  < 0.01), and in the model + CSL + oeNLRP3 group compared to the model + CSL + oeNC group (*p*  < 0.01) (Figure 5B). Micro-CT analysis revealed severe cartilage damage in the model group, with elevated BV/TV and BMD values (both *p*  < 0.01), which were attenuated by CSL treatment (*p*  < 0.01). However, cartilage degeneration was exacerbated in the model + CSL + shSIRT2 and model + CSL + oeNLRP3 groups, with BV/TV and BMD values increased compared to their respective controls (*p*  < 0.01) (Figure 5C). H&E staining showed reduced cartilage thickness and chondrocyte numbers in the model group (*p*  < 0.01), but improved in the model + CSL group (*p*  < 0.01). In contrast, cartilage thickness and cell number were markedly decreased in the model + CSL + shSIRT2 and model + CSL + oeNLRP3 groups compared to their corresponding controls (*p*  < 0.01) (Figure 5D). Western blot analysis revealed upregulation of NLRP3, cleaved-caspase-1, cleaved-IL-1β, and GSDMD-N in the model group compared to the sham group (*p*  < 0.01 for all), and suppressed in the model + CSL group (*p*  < 0.01). These proteins were significantly elevated again in the model + CSL + shSIRT2 and model + CSL + oeNLRP3 groups compared to their respective control groups (*p*  < 0.01) (Figure 5E). Immunohistochemical staining showed increased NLRP3 expression and decreased SIRT2 expression in the model group. CSL treatment reversed this pattern by reducing NLRP3 and increasing SIRT2 expression. However, NLRP3 expression increased and SIRT2 expression decreased in the model + CSL + shSIRT2 group. In the model + CSL + oeNLRP3 group, NLRP3 expression increased while SIRT2 expression remained unchanged (Figure 5F). ELISA analysis showed that, compared to the sham group, proinflammatory cytokines IL-1β (*p*  < 0.01) and TNF-α (*p*  < 0.01) were significantly elevated in the model group, while anti-inflammatory cytokines IL-4 and IL-10 showed no significant changes (*p*  > 0.05). Additionally, SAA (*p*  < 0.01) and CRP (*p*  < 0.01) levels were increased. The model + CSL group exhibited significantly reduced IL-1β, TNF-α, SAA, and CRP levels (*p*  < 0.01), with increased IL-4 and IL-10 (*p*  < 0.01). Compared to the model + CSL + shNC group, the model + CSL + shSIRT2 group showed higher IL-1β, TNF-α, SAA, and CRP levels (*p*  < 0.01), and lower IL-4 and IL-10 levels (*p*  < 0.01). Similarly, compared to the model + CSL + oeNC group, the model + CSL + oeNLRP3 group displayed elevated IL-1β, TNF-α, SAA, and CRP (*p*  < 0.01), and decreased IL-4 and IL-10 (*p*  < 0.01).

In summary, the results above demonstrate that CSL can upregulate SIRT2 to mediate the deacetylation inactivation of NLRP3, thereby, inhibiting chondrocyte death and preventing the progression of OA inflammation.

## 4. Discussion

OA is a globally prevalent joint disease characterized by the progressive destruction of articular cartilage, inflammation, joint effusion, and pain [56]. With an aging population and increasing obesity rates, the incidence of OA has risen significantly. CSL is a natural pentacyclic triterpenoid compound extracted from the traditional Chinese medicine *Tripterygium wilfordii*, known for its significant anti-inflammatory, antioxidant, and immunomodulatory effects [19, 20]. In recent years, CSL has gained widespread attention in the scientific community due to its broad biological activities and has demonstrated potential therapeutic effects in various disease models [20, 22].

This study employed network pharmacology and bioinformatics methods, combined with in vitro and in vivo experiments, to systematically investigate the mechanisms of action of CSL in the treatment of OA. Our findings indicate that CSL upregulates SIRT2 expression, which mediates the deacetylation and inactivation of NLRP3, reducing pro-inflammatory cytokines and promoting IL-4 and IL-10 expression. CSL inhibits chondrocyte pyroptosis and halts OA inflammation. This discovery provides a crucial molecular basis for CSL as a potential therapeutic agent for OA. In comparison to prior studies, our study not only elucidates the specific mechanism of action of CSL but also validates its potential in OA treatment through multilevel experiments.

In this study, although ZC3H12A, BCL2, and EGFR were identified as key candidate genes in the initial network pharmacology screening, molecular docking results revealed that their binding energies with CSL exceeded −10 kJ/mol, failing to meet the threshold for strong molecular interaction. Consequently, they were excluded from the final list of potential targets. Nonetheless, these genes remain biologically relevant in the inflammatory pathways of OA and may participate in other regulatory processes. ZC3H12A is implicated in immune-inflammatory regulation, BCL2 in apoptotic pathways, and EGFR in cell proliferation and tissue repair—all of which have well-established roles in OA pathogenesis. The responsiveness of these targets to CSL remains unclear and warrants further investigation. Future experiments at the cellular level are planned to assess the expression changes of ZC3H12A, BCL2, and EGFR upon CSL treatment, thereby, supplementing validation of these potential “negative results” and enriching the interpretation of the target network.

Previous studies have suggested that CSL possesses broad anti-inflammatory and immune-modulating effects; however, its specific mechanism in OA remains unclear [57, 58]. Our study reveals that CSL exerts its effects in OA through the SIRT2/NLRP3 axis. Oxidative stress and ROS significantly regulate NLRP3 inflammasome activation by inducing NLRP3 acetylation. CSL exerts antioxidant effects by activating NRF2, reducing ROS levels, and inhibiting NLRP3 activation. NLRP3 activation triggers the release of pro-inflammatory cytokines IL-1β and IL-18, amplifying inflammation and pyroptosis. The NF-κB pathway, activated downstream of NLRP3, further exacerbates inflammation in OA. CSL upregulates SIRT2 expression, directly regulating NLRP3 acetylation and modulating oxidative stress and NF-κB pathways, thus, reducing inflammatory factor release and alleviating OA inflammation. Previous studies have employed liposomal nanotechnology to deliver Ginkgo flavone glycosides, demonstrating that this approach can activate SIRT1 and alleviate the pathological processes of diabetic cardiomyopathy by modulating oxidative stress and inflammatory responses [59]. Although SIRT1 and SIRT2 belong to the same sirtuin family, their regulatory targets and biological contexts differ. Our study reveals that in OA, SIRT2 plays a more pivotal role in regulating NLRP3 inflammasome activation and downstream pyroptosis, further distinguishing the mechanism of CSL from other sirtuin-targeted therapeutic strategies.

NLRP3 is an important inflammasome widely studied in various inflammatory diseases [60]. Its excessive activation is closely related to the onset of various inflammatory and autoimmune diseases [61, 62]. In OA, NLRP3 activation induces chondrocyte pyroptosis, worsening joint inflammation and damage [62–64]. SIRT2, as an NAD^+^-dependent deacetylase, has been shown to play an important role in various cellular processes. However, its specific role in OA is still unclear [65, 66]. Through co-immunoprecipitation, we confirmed the interaction between SIRT2 and NLRP3, and validated the regulatory role of SIRT2 in NLRP3 activation through in vitro and in vivo experiments, providing new evidence for SIRT2 as a therapeutic target for OA. Specifically, SIRT2 inhibits NLRP3 activation through deacetylation, thereby, reducing inflammation and pyroptosis. This finding aligns with previous studies on SIRT2 in other inflammatory diseases, further confirming its potential value as a key regulator of inflammation in OA [54, 55, 67–69].

Cytokines are key components in the pathogenesis of OA, acting on both chondrocytes and immune cells to trigger the release of inflammatory mediators and proteases [70]. Pro-inflammatory cytokines like IL-1β and TNF-α degrade the extracellular matrix, induce chondrocyte apoptosis, and activate MMPs, contributing to joint cartilage degeneration [71]. Therefore, inhibiting the release of these inflammatory factors or blocking their downstream effects is critical for the prevention and treatment of OA. IL-4 and IL-10 have anti-inflammatory effects and improve OA progression [70]. They inhibit the production of pro-inflammatory cytokines including TNF-α, IL-1β, and IL-6 [72, 73]. Previous studies have shown that IL-4 inhibits MMP secretion, suppressing the degradation of proteoglycans in joint cartilage [74]. IL-4 alone or in combination with IL-10 has been shown to have apoptosis-inhibiting effects on chondrocytes and fibroblast-like synovial cells [70]. Our findings indicate that LPS induced mRNA expression of pro-inflammatory mediators in macrophages did not significantly alter IL-4 and IL-10 levels within 3 days, and no notable changes in serum IL-10 were observed in the OA rat model. CSL inhibits the secretion of pro-inflammatory cytokines IL-1β and TNF-α while promoting IL-4 and IL-10 production, and this anti-inflammatory effect is dependent on SIRT2. CSL has been shown to increase IL-10 expression in an acute ischemic stroke model [75]. However, there are currently limited studies directly investigating the interaction between CSL and IL-4 and IL-10, and further investigations are needed to explore the specific mechanisms and clinical application value.

This study integrates in vitro and in vivo experiments to comprehensively evaluate the mechanisms of action of CSL in OA. In vitro experiments validate CSL's regulatory effect on the SIRT2/NLRP3 signaling pathway in cell models, while in vivo experiments confirm the therapeutic effects of CSL through a rat model. This comprehensive experimental design enhances the credibility and application prospects of the research findings, laying a foundation for further clinical studies. Through behavioral and histological analyses in the rat model, we further affirm the therapeutic effects of CSL.

This study sheds light on the mechanism of action of CSL in OA, providing a novel therapeutic target and strategy. In particular, we discovered that CSL upregulates SIRT2 expression, mediating the deacetylation and inactivation of NLRP3, thus, inhibiting chondrocyte pyroptosis and effectively halting the inflammation progression of OA. The elucidation of this mechanism not only enriches our understanding of the pathophysiology of OA but also provides a theoretical basis for the development of new and effective therapeutic drugs. Clinically, this finding holds promise for translating into a new treatment approach, offering OA patients a novel treatment option based on natural compounds, potentially reducing the side effects of current treatments, improving treatment efficacy, and ultimately enhancing patients' quality of life and prognosis.

The OA rat model used in this study was established via intra-articular injection of MIA, selected for its pathological similarity to human OA. MIA inhibits glyceraldehyde-3-phosphate dehydrogenase (GAPDH), leading to disrupted chondrocyte energy metabolism and mitochondrial dysfunction, which mimics core features of human OA such as cartilage degradation, synovial inflammation, and pain [37]. Moreover, the MIA model induces stable cartilage damage and inflammation within 2–4 weeks, making it suitable for rapid pharmacological evaluation [38]. Since this study focused on the SIRT2/NLRP3-mediated inflammatory and pyroptotic mechanisms, the MIA model was appropriate given its ability to activate NLRP3 inflammasomes effectively. Nevertheless, the MIA model has intrinsic limitations due to its chemically induced pathology, which does not encompass other etiologies of OA such as mechanical stress, age-related degeneration, or obesity. Compared to spontaneous models (e.g., STR/Ort mice) or traumatic models (e.g., ACL transection), the MIA model lacks the progressive cartilage repair and remodeling process, which may underestimate disease complexity [28]. Future studies will incorporate large animal models (e.g., dogs or goats) that better replicate human OA pathogenesis to further validate the generalizability of our findings.

Based on previously published studies confirming its safety and anti-inflammatory efficacy, CSL was administered at 20 μg/mL via intra-articular injection [38]. Preliminary CCK-8 assays confirmed that 1 μM CSL does not exhibit significant cytotoxicity in chondrocytes (viability >85%) while effectively suppressing inflammatory cytokine release. Nonetheless, future investigations will explore the toxicological risks associated with higher doses, emphasizing the need for careful dosage monitoring in clinical translation [75]. Due to time and funding constraints, commonly used OA treatments were not included as positive controls in this study—an acknowledged limitation. In subsequent research, we will incorporate conventional therapeutics to facilitate comparative efficacy analysis with CSL.

While this study has made significant progress in elucidating the influence of the SIRT2/NLRP3 signaling axis in OA, several limitations should be acknowledged. First, our findings on pyroptosis were primarily based on the expression of key molecular markers (e.g., NLRP3, cleaved GSDMD, and Caspase-1). Although these changes strongly suggest pyroptosis in chondrocytes, morphological evidence such as membrane rupture and blebbing was not directly observed due to the absence of ultrastructural analyses. Future studies incorporating transmission electron microscopy and live-cell imaging are needed to confirm these features. Second, this study mainly focused on the SIRT2/NLRP3 pathway and did not explore other potentially relevant mechanisms of CSL in OA. Third, although both in vitro and in vivo experiments support the therapeutic potential of CSL, the long-term effects and specific molecular mechanisms remain to be fully understood. Additionally, the animal and cell models employed may not fully replicate the complex pathophysiology of human OA, underscoring the need for more advanced models and clinical investigations. Future studies should explore the multi-target effects of CSL at various pathological stages of OA and elucidate the crosstalk between the SIRT2/NLRP3 axis and other inflammatory pathways to identify novel therapeutic strategies. Stepwise clinical trials are also warranted to assess the efficacy and safety of CSL in diverse patient populations, ultimately paving the way for its clinical translation.

## 5. Conclusion

**Figure 6:**
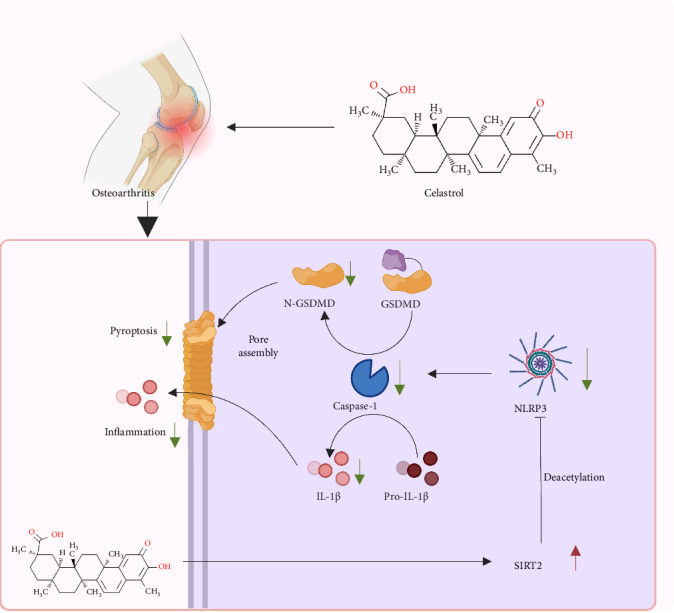
CSL inhibits OA through regulating the SIRT2/NLRP3 axis molecular mechanism.

In conclusion, through the integrated application of network pharmacology and bioinformatics approaches, we systematically identified and validated the key targets and signaling pathways of CSL, elucidating its molecular mechanism in inhibiting the inflammatory progression of OA (Figure 6). Our findings suggest that CSL modulates the SIRT2/NLRP3 axis, upregulating SIRT2 expression, inhibiting NLRP3 acetylation and activation, and reducing pro-inflammatory cytokines. These actions consequently lower levels of cell apoptosis and pyroptosis, ultimately exerting anti-inflammatory and chondroprotective effects. This discovery enhances our understanding of OA pathogenesis and supports the development of novel therapeutic strategies.

## Data Availability

The data that support the findings of this study are available from the corresponding author upon reasonable request.

## Supporting Information

Supporting Information 1 Figure S1. Screening workflow of potential targets through which celastrol modulates the inflammatory progression of osteoarthritis.

Supporting Information 2 Table S1. Topological coefficients of 15 key target points. Table S2. Functional enrichment frequency of 15 key target points. Table S3. The lowest binding energy of docking for five candidate target molecules.

Supporting Information 3 Figure S2. Identification of RCs.
