# Supplementary figures and images for: Mechanistic Insights Into Celastrol's Anti-Pyroptosis Effects in Osteoarthritis via SIRT2 Upregulation
Source: Mediators Inflamm. 2025 Sep 8;2025:5676471. doi: 10.1155/mi/5676471 (PMC12436004; doi:10.1155/mi/5676471)

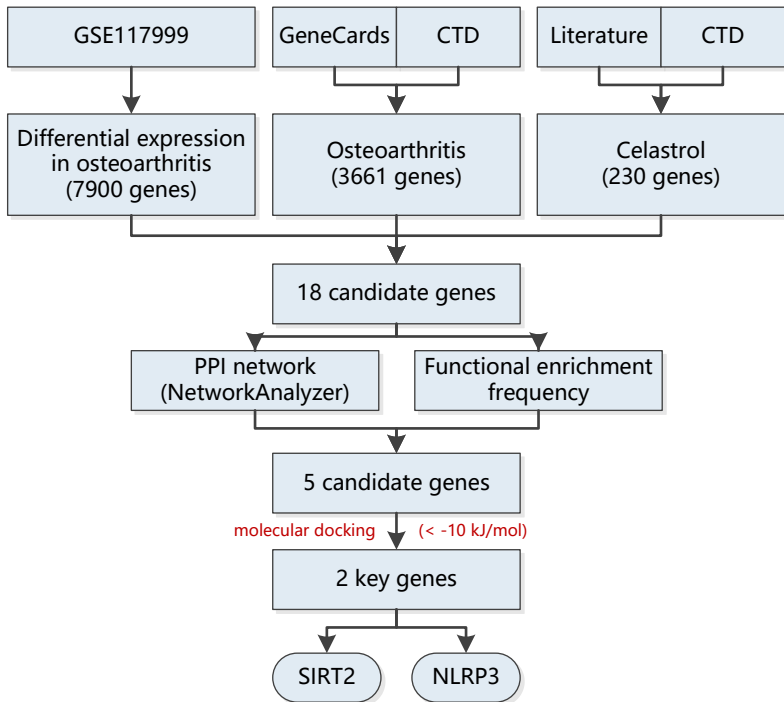

Supplement: Supporting Information 1 — Figure S1. Screening workflow of potential targets through which celastrol modulates the inflammatory progression of osteoarthritis. [file 5676471.f1.pdf]

DAPI

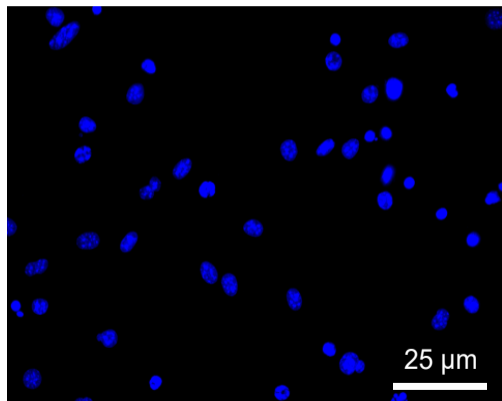

Collagen II

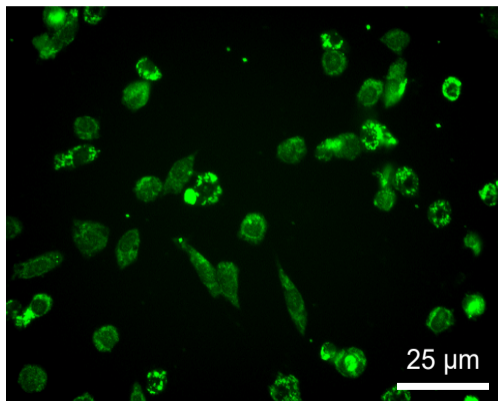

Merge

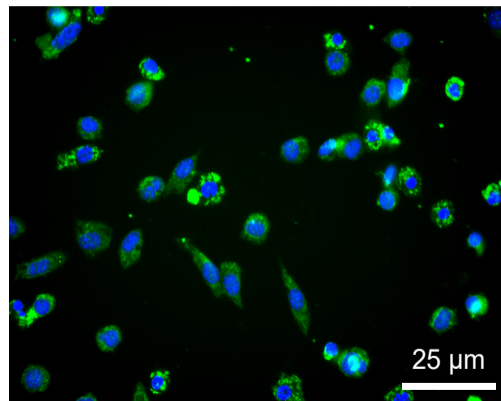

Supplement: Supporting Information 3 — Figure S2. Identification of RCs. [file 5676471.f3.pdf]
