# Supplementary material for: Mechanistic Insights Into Celastrol's Anti-Pyroptosis Effects in Osteoarthritis via SIRT2 Upregulation
Source: Mediators Inflamm. 2025 Sep 8;2025:5676471. doi: 10.1155/mi/5676471 (PMC12436004; doi:10.1155/mi/5676471)
Supplement: Supporting Information 2 — Table S1. Topological coefficients of 15 key target points. Table S2. Functional enrichment frequency of 15 key target points. Table S3. The lowest binding energy of docking for five candidate target molecules. [file 5676471.f2.docx]

**Table S1. Topological coefficients of 15 key target points.**

| **Gene** | **Topological Coefficient** |
| --- | --- |
| ZC3H12A | 0.911458333 |
| DDIT4 | 0.567073171 |
| TLR7 | 0.54885756 |
| GPT | 0.535333807 |
| SIRT2 | 0.532971996 |
| MMP1 | 0.526794742 |
| MMP3 | 0.514811198 |
| NLRP3 | 0.505371094 |
| CASP9 | 0.503240564 |
| AXIN2 | 0.494545455 |
| WIF1 | 0.486102133 |
| BAG3 | 0.472532815 |
| CYCS | 0.446555301 |
| BCL2 | 0.434108527 |
| EGFR | 0.405445117 |

**Table S2. Functional enrichment frequency of 15 key target points.**

| **Gene** | **Freq** |
| --- | --- |
| ZC3H12A | 87 |
| BCL2 | 72 |
| NLRP3 | 52 |
| SIRT2 | 47 |
| EGFR | 45 |
| CASP9 | 41 |
| DDIT4 | 34 |
| AXIN2 | 27 |
| MMP3 | 22 |
| TLR7 | 21 |
| BAG3 | 20 |
| OPRK1 | 18 |
| MMP1 | 14 |
| CYCS | 11 |
| WIF1 | 4 |
| GPT | 2 |
| SLTM | 1 |

**Table S3. Lowest binding energy of docking for 5 candidate target molecules.**

| **Gene** | **PDB** | **Lowest Binding Energy (kj/mol)** |
| --- | --- | --- |
| ZC3H12A | 6q3v | -7.38 |
| BCL2 | 6zx7 | -9.77 |
| NLRP3 | 4b5o | -10.63 |
| SIRT2 | 4bn4 | -13.26 |
| EGFR | 8a27 | -9.51 |
